# Harvest Age Effect on Phytochemical Content of White and Black Glutinous Rice Cultivars

**DOI:** 10.3390/molecules24244432

**Published:** 2019-12-04

**Authors:** Kawintra Tamprasit, Natthida Weerapreeyakul, Khaetthareeya Sutthanut, Wipawee Thukhammee, Jintanaporn Wattanathorn

**Affiliations:** 1 Graduate School (in the program of Aesthetic Sciences and Health), Faculty of Pharmaceutical Sciences, Khon Kaen University, Khon Kaen 40002, Thailand; kawintra_pop@hotmail.com; 2 Division of Pharmaceutical Chemistry, Faculty of Pharmaceutical Sciences, Khon Kaen University, Khon Kaen 40002, Thailand; khaesu@kku.ac.th; 3 Human High Performance and Health Promotion (HHP&HP) Research Institute, Khon Kaen University, Khon Kaen 40002, Thailand; meewep@gmail.com (W.T.); jinwat05@gmail.com (J.W.); 4 Department of Physiology, Faculty of Medicine, Khon Kaen University, Khon Kaen 40002, Thailand

**Keywords:** rice, rice sprout, white glutinous rice, black glutinous rice

## Abstract

Many studies have been conducted on the bioactive compounds of rice seeds, however, there is limited information on the bioactive compounds of rice sprouts. This study focused on the age effect on the phytochemical content of white and black glutinous rice sprouts harvested between 5 and 25 days old. We assessed yield, total phenolic content, total flavonoid content, total anthocyanin content, total chlorophyll content, and proximate analysis. HPLC results identified protocatechuic acid, vanillic acid, and rutin in the sprouts of both cultivars, ranging between 0.56–1.58, 0.65–7.69, and 0.47–1.68 mg/g extract, respectively. The amount of bioactive compounds and proximate compositions in black glutinous rice were generally higher than white glutinous rice in an age-dependent manner (*p* < 0.05). At 5–7 days, black glutinous rice contained the highest total anthocyanin content, while white glutinous rice contained the highest total phenolic content and total flavonoid content (*p* < 0.05). High total chlorophyll content was initially detected in white glutinous rice at a younger age than black glutinous rice (*p* < 0.05), while total chlorophyll content in both cultivars was not significantly different. Our study confirms the presence of phytoconstituents in the rice sprouts of white and black glutinous rice and their potential as functional foods and for being further development as natural health products.

## 1. Introduction

Rice is a major agricultural crops in many parts of the world. Asian rice (*Oryza sativa*) is one of the most important commodities in the agriculture sector in Asian countries. According to the Food and Agriculture Organization (FAO), Asia has the highest paddy rice production followed by the Americas, Africa, Europe and Oceania [1]. Based on a FAO report [1], global paddy rice production has been constantly increasing. Rice utilization in 2017–2018 was an estimated 503.9 million tonnes (milled basis) [1]. The productivity of paddy rice depends on weather, seed type, and soil conditions [2], so global warming or climate change will have a large impacts on rice production and rice availability around the world [3]. Estimated world paddy rice production by FAO in 2017 only slightly exceeded the record in 2016 by 0.6 percent, possibly because of a disruption in the main crop cycle by floods or ongoing drought [1,4]. The small increase in paddy rice production was offset in some countries by secondary crops [1]. To increase the competitiveness in the agriculture sector, the other parts of rice could be exploited. Several studies have explored the beneficial uses of all parts of the rice plant and developing innovative rice products and zero waste [5,6]. For instance, the combustion of rice husks and rice straw—by-products of rice production and milling—was used as biomass for developing high quality biogenic silica [5]: the eco-friendly technology used biomass as a sustainable energy source to produce zero waste [5,6]. The major and common edible parts of rice are grain, husked rice, and rice bran over against the less edible young rice leaves [7]. The great potential of rice includes not only its being consumed as food and beverage, but also its use in promoting health and wellness through dietary supplements and cosmetic products [7,8,9,10,11,12].

An example of a healthy food taken from the sprout is wheatgrass. Wheatgrass comprises many bioactive constituents—including chlorophyll, iron chelators, bioflavonoids, vitamins C and E, minerals, amino acids, and vital enzymes (i.e., superoxide dismutase and cytochrome oxidase) [13,14]. Wheatgrass has been proposed as an adjuvant in anti-allergic and anti-asthmatic treatment, reducing myelotoxicity induced by chemotherapy [15]. Based on clinical studies, reported the health benefits of wheatgrass included: detoxification of bisphenol A in young women [14,16], treatment of an active distal ulcerative colitis [17], reduction of oxidative stress in healthy subjects [18], and reduction of blood transfusion requirements in thalassemia [14,19,20].

One study on the rice leaf as an alternative health food reported on seven white and seven colored Thai rice cultivars [21]. The bioactivity study reported on the rice leaf juices collected at jointing stage as compared to wheatgrass juice [21]. It was revealed that the antioxidant capability—based on 2,2–diphenyl–1–picrylhydrazyl, ferric reducing antioxidant power, β–carotene bleaching, thiobarbituric acid reactive substances assays, the total phenolic content (TPC), total monomeric anthocyanin content (TMAC)—of the juices of colored (purple) rice sprout exhibited greater antioxidant capability than the white rice and wheat. The colored rice ‘Kum Doisaket’ exhibited the highest antioxidant activity and greatest DNA protective effect in all assays in a dose-dependent manner. These activities were attributed to the high level of anthocyanins of this colored rice. These authors suggested the possibility of developing functional foods from colored rice sprout [21].

The information regarding the potential of rice sprout vis-à-vis the presence of active compounds contributed to the health benefit is limited. In the present study, the nutrition values, phenolics and flavonoids contents of rice RD6 and black glutinous (BGR) sprouts were evaluated. These two rice cultivars represent the most common Thai native white and black grain glutinous rice. They are widely consumed throughout northeastern Thailand and other Asian countries. The effect of harvest time was assessed with respect to yields, total phenolic content, total flavonoid content, total anthocyanin content, total chlorophyll content, and proximate compositions of rice sprouts. The rice sprouts were harvested between 5 and 25 days of age, mimicking agricultural commercial cycles. This research may lead an increase in value-added of rice products and market access with follow-on economic benefits for farmer.

## 2. Results

### 2.1. Extraction Yield

**Figure 1:**
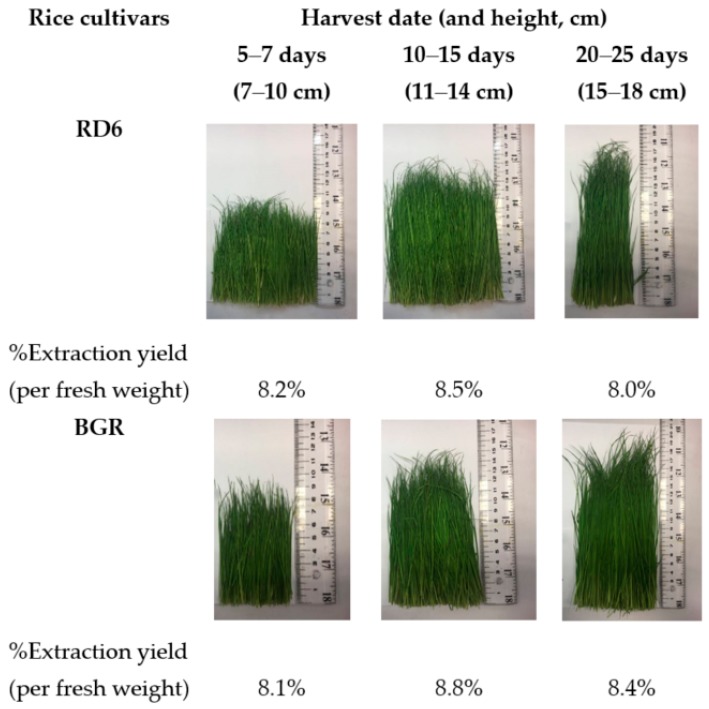
RD6 and BGR at different harvest date and yield.

The rice sprout of two cultivars (RD6 and BGR) were collected at different height ranges which was correlated to age ranges (Figure 1). The extraction yield was calculated from the percentage of weight of extract per weight of fresh sprout. This value reflects the extraction conditions. The respective extraction yield of the dry extract obtained from fresh RD6 and BGR sprout ranged between 8.0 and 8.8% (Figure 1). The extraction yield of BGR was higher than that of the RD6. Both cultivars had a maximum %extraction yield at age 10–15 days (height range, 11–14 cm).

### 2.2. HPLC Analysis for Phenolic and Flavonoid Contents

The respective phenolic and flavonoid contents was identified using HPLC analysis based on three different wavelengths. Ten standard compounds were used for identification, including (i) hydroxybenzoic acids (viz., gallic acid, protocatechuic acid, *p*–hydroxybenzoic acid, vanillic acid, syringic acid), (ii) hydroxycinnamic acids (viz., chlorogenic acid, caffeic acid, *p*–coumaric acid, ferulic acid), and (iii) flavonoids (viz., rutin). Compounds in the extracts were identified by comparing the retention times and UV spectra with those of authentic compounds (Supplementary Materials, Figure S1), and confirmed by co–injecting the sample and the target standard compound. The retention time at each detected wavelength from the HPLC chromatograms of phenolics and flavonoids in the rice sprout extracts from RD6 and BGR cultivars are shown in Table 1. 

Ten standard phenolic and flavonoid compounds were selected for qualitative and quantitative analysis using HPLC as they are commonly found in various plants and have been previously studied. Notwithstanding, only three compounds were identified in our study―protocatechuic acid (2), vanillic acid (5), and rutin (10); the lack of detection of other compounds could be due to the natural diversity of these compounds in plants (i.e., in the current study) and/or a difference in growing conditions in the location of collection (i.e., in the previous study). According to the peak height, the amount of protocatechuic acid, vanillic acid, and rutin in the two cultivars is different and related to age (Table 2). The respective phenolic and flavonoid contents in the rice sprout extracts was calculated from the peak height compared to the standard compounds. Protocatechuic acid content was the highest in RD6 at 5–7 days (1.58 ± 0.12 mg/g dry extract). Vanillic acid content was the highest in BGR at 5–7 days (7.69 ± 0.58 mg/g dry extract). Rutin was found in the two cultivars with the highest content being in RD6 at 5–7 days (1.68 ± 0.11 mg/g dry extract). Phenolic compounds with a hydroxybenzoic acid structure were predominantly detected in BGR and with a higher content than the flavonoid compound. The total detected phenolic contents in BGR was higher than in RD6 at the early harvesting date. By contrast, the detected flavonoids had a higher quantity in RD6 than in BGR at the early harvest date. Since extraction method and HPLC condition can affect the presence and content of compounds in the extracts, and since there were other unidentified peak, total phenolic and flavonoid contents were also determined. 

### 2.3. Total Phenolic Content (TPC)

The phenolic content (TPC) in the sprout extract of RD6 and BGR are shown in Table 3. TPC in each cultivar was significantly different at different harvest dates (*p* < 0.001). TPC in RD6 decreased as age increased. RD6 at 5–7 days had the highest TPC which was higher than the content at 10–15 days and 20–25 days. The TPC in RD6 ranged between 29.1 and 44.8 mg GAE/g of extract. While the TPC in BGR ranged between 24.9 and 31.9 mg GAE/g of extract. The highest TPC in BGR was found at 10–15 days which was higher than the content of 5–7 days and 20–25 days. In summary, TPC in RD6 was significantly higher than that of BGR.

### 2.4. Total Chlorophyll Content (TCC)

A previous study reported a non–significantly difference between the chlorophyll content as determined by spectrophotometer and HPLC analysis [22]. The current study, therefore, determined chlorophyll using a microplate reader as it is a relatively simple and fast technique. The extracts were dissolved in methanol as it is a good extractant for chlorophyll [23]. Based on UV absorbance scanning at the specific wavelength, both RD6 and BGR cultivars had major peaks for chlorophyll A (400–450 and 650–700 nm) and chlorophyll B (590–600 nm). Our results showed the difference in total chlorophyll contents (TCC) in the sprout extract of RD6 and BGR (Table 3). The TCC of RD6 at different ages ranged between 34.6 ± 2.1 and 40.5 ± 2.2 mg/g extract. The highest TCC in RD6 was found at the age of 5–7 days over against other ages (*p* < 0.001). The TCC in BGR ranged between 19.3 ± 1.8 and 40.8 ± 0.8 mg/g extract with the highest TCC at age 10–15 days.

### 2.5. Total Anthocyanin Content (TAC)

Total anthocyanin content (TAC) was measured according to the pH differential method. Results in Table 3 showed that the BGR possessed a higher TAC than RD6. The total anthocyanin of RD6 increased with ages albeit not statistically different (range, 0.01 ± 0.001 to 0.15 ± 0.001 mg C_3_GE per g dry extract). RD6 at 20–25 days had the highest TAC over against the other ages. TAC in BGR ranged between 0.45 ± 0.001 and 22.13 ± 0.001 mg C_3_GE per g dry extract. The highest TAC in BGR was at 5–7 days, which was higher than 10–15 days (*p* < 0.001) and 20–25 days (*p* < 0.001).

### 2.6. Total Flavonoid Content (TFC)

The respective total flavonoid contents of the RD6 and BGR extract is presented in Table 3. The TFC of the RD6 and BGR cultivars ranged between 16.3 ± 0.5 and 34.2 ± 3.4 mg quercetin equivalent (QE)/g of extract. RD6 at 5–7 days showed a slightly higher flavonoid content than at 10–15 days (*p* = 0.696) and significantly higher than at age 20–25 days (*p* < 0.001). BGR at 10–15 days (30.3 ± 0.3 mg QE/g extract) and 20–25 days (29.2 ± 0.7 mg QE/g extract) showed no significantly difference in TFC (*p* = 0.935), but a significantly difference at age 5–7 day (21.5 ± 1.4 mg QE/g extract) (*p* < 0.001).

### 2.7. Proximate

The proximate analysis of the RD6 and BGR sprouts at different ages are presented in Table 3. The fresh weight (100 g) of the rice sprouts from both RD6 and BGR had a total ash between 2.86 and 5.78 g, moisture ranged from 64.72 and 79.11 g, fat 0.32–1.2 g, protein 3.38–5.99 g, carbohydrate, 12.22–24.54 g, and energy 73.44–124.0 Kcal. The rice sprouts of both cultivars at 10–15 days were selected for dietary fiber determination as this age showed the highest contents of compounds and nutrients, while the older sprouts was hard to chew. The respective dietary fiber in RD6 and BGR fresh sprouts was 11.98 and 16.92 g/100 g fresh weight. BGR has 1.4 times higher dietary fiber than RD6.

## 3. Discussion

Total phenolic content (TPC) was determined by using the Folin–Ciocalteu assay. The compounds in the extracts were determined by HPLC and compared with the standard peak. Total chlorophyll content was determined by UV–Vis spectrophotometrically. Total anthocyanin content was determined by the pH differential method. Total flavonoid content (TFC) was determined by Dowd method. The proximate tests were conducted to investigate the ash, moisture, fat, protein, carbohydrate, energy, and dietary fiber content. These colorimetric methods represent the most adopted protocols for the quantification of TPC, TFC, and TAC in food, vegetable, and herbal samples [7]. 

High TPC and TFC levels in RD6 occurred at an early age but decreased with plant age. With respect to the R^2^ values for RD6 (Supplementary Materials, Table S1), the pairs of harvest date and TPC (R^2^ = 0.822), and pair of TFC and TPC (R^2^ = 0.931) are linearly related to harvest date. While TPC and TFC in BGR gradually increased from early age to middle age albeit not linearly correlated to harvest date. TAC was significantly higher in BGR; especially at an early age but declined with age. Concerning the three different ages of rice (viz., at 5–7, 10–15 and 20–25 days), TPC in RD6 sprouts was significantly greater than BGR (16.7, 9.6 and 4.2 mg GAE, respectively). A higher TFC in RD6 was also observed (12.7, 2.3 at 5–7 and 10–15 days) than BGR sprouts, while the latter showed a higher anthocyanin (22.07, 0.44 and 0.56 mgC_3_GE, respectively) than RD6 (*p* < 0.05). With respect to the R^2^ values of BGR (Supplementary Materials, Table S1), it is clear that pairs of TCC and TAC (R^2^ = 1.000), TCC and TFC (R^2^ = 0.962), and TAC and TFC (R^2^ = 0.948) are linearly related to the harvest date.

According to the partial correlation analysis of the data for BGR (Supplementary Materials, Table S2), there are a strong and significant negative correlations between harvest date and TAC (−0.880), and a strong and significant positive correlation between harvest date and TCC (0.863), and TFC (0.791) (*p* < 0.05). No significant negative correlation (*p* = 0.292) was found between harvest date and TPC (−0.396). A strong and significant negative correlation between TCC and TAC (−1.000), and between TAC and TFC (−0.974) were observed (*p* < 0.001). Moreover, a strong and significant positive correlation was found between TCC and TFC (0.981) (*p* < 0.001). All other correlation coefficients were shown to be weak (between 0.107 and 0.210) with no statistical significance (*p* > 0.05). Our data suggest that in BGR, the harvest date affected the content of anthocyanins, chlorophyll, and flavonoids but not phenolics.

Based on the data analysis of RD6, there are a strong and significant negative correlation between harvest date and TPC (−0.944), TCC (−0.735) as well as TFC (−0.883), and a strong significant positive correlation between TFC and TPC (0.965) (*p* < 0.05). All other correlation coefficients were shown to be weak to moderate (between 0.045 and 0.518) with no statistical significance (*p* > 0.05). The data suggest that in RD6, the harvest date affected the content of phenolics, chlorophyll, and flavonoids but not anthocyanins.

Khanthapok et al. reported on the TPC contents in rice sprouts. The TPC of juices from colored rice sprouts ranged between 1.9 and 4.3 mg GAE/g dry extract [21], while the TPC of juices from white rice sprouts ranged between 1.50 and 2.14 mg GAE/g dry extract, and the TPC of wheatgrass juice was 2.91 ± 0.1 mg GAE/g dry extract [21]. A similar trend was observed but in the rice brans from RD6 and black rice bran [24]. The TPC in the rice bran of RD6 was 1.96 ± 0.04 (mg GAE/g dry extract), which was less than that in the black rice bran (6.65 ± 0.93 (mg GAE/g dry extract). Our results by comparison revealed a higher TPC in RD6 sprouts than the BGR (colored rice).

As chlorophyll possesses a structure similar to that of hemoglobin, it has been reported to regenerate or act as a substitute for hemoglobin in hemoglobin deficiency conditions [20]. TCC was previously found in the drinks made from barley sprouts, wheat, and three cultivars of Thai rice (i.e., Jasmine rice, ‘Sukhothai 1’, and ‘Sukhothai 2’) [25]. The TCC ranged between 82 and 958 μg/200 mL or between 0.07 and 0.8% of solids [25]. Barley sprout drink contained more chlorophyll content than drinks made from the sprouts of Jasmine rice, ‘Sukhothai 2’, wheat, and ‘Sukhothai 1’, respectively. The chlorophyll contents were lesser than those of wheatgrass juice (70% of solids) that was proposed for clinical treatment of hemoglobin deficiency treatment or thalassemia major [14,19]. A tablet of wheatgrass was also formulated (500 mg per tablet) and was reportedly clinically beneficial after a year of use as an adjuvant for treatment of thalassemia and hemolytic anemia [20]. Relatedly, chlorophyll was used to reduce acne [26] and for anti-aging [27].

TACs were previously reported in the juice of colored rice sprouts at 4.42 mg C_3_GE/g DE [21]. A small amount of anthocyanin has been reported in non–pigmented cultivars; the contents of anthocyanin in white wheat grain cultivar was 7 μg/g dry extract [28]. Another study showed that non–pigmented rice bran had lesser anthocyanin content (12 ± 3.6 μg/g dry extract) than the pigmented rice bran (102–3594 μg/g dry extract) [29]. TAC in rice sprouts from African rice cultivars ranged between 0.001 and 0.003 mg/g dry weight [30]. Our results are consistent with previous studies that a higher TAC was found in the colored rice compared to the white rice.

Flavonoids are the naturally occurring antioxidants that provide many clinical benefits such as management of inflammatory bowel disease and as a general detoxifier. The TFC was determined by spectrophotometry in five varieties of African rice sprouts and ranged between 0.014 and 0.043 mg/g dry weight by using quercetin as the standard [30]. Hence, Thai rice at the age collected for this study had a higher TFC than African rice sprouts.

Previous research into the proximate analysis of rice sprout was lacking. Two studies determined the proximate analysis of rice bran. White rice bran from Indonesia was reported to contain 11.22% moisture, 14.39% fiber, 42.87% carbohydrate, 10.74% protein, 11.42% fat, and 8.23% ash [31]. RD6 rice bran comprised 16.96 ± 0.41% fat, 12.07 ± 0.23% protein, 42.54 ± 0.62% carbohydrate, 11.77 ± 0.49% fiber, and 10.78 ± 0.33% ash. BGR rice bran contained 15.85 ± 0.47% fat, 13.27 ± 0.05% protein, 45.06 ± 2.11% carbohydrate, 12.68 ± 0.79% fiber, and 9.72 ± 0.12% ash [24]. In previous research, the rice bran of RD6 and BGR were reported to contain more carbohydrate, protein, lipid, and ash [24] than that reported in our study. To compare, the rice sprouts of BGR in the present study contained greater fiber than that reported for rice bran [24,31].

Many studies of bioactive compounds have been conducted on rice seeds whereas information on the bioactive compounds in rice sprout is scarce. Rice is a good source of vitamins B1, vitamin B2, vitamin B3 and vitamin B6, albeit there is a wide variation is observed among cultivars [2]. Removing the husk yields whole grain or brown rice. Further polishing removes the bran and germ resulting in white rice. Whole grain rice has a high nutritional value [7] as it retains the bran layer containing many vitamins, minerals, and fibers [2]. Different anthocyanin pigments of the outer layer of whole grain rice give rise to the colors (e.g., black, purple, or red) [7]. Anthocyanins—a water-soluble pigment—contributes to free radical scavenging, antioxidant capacity, and several well-documented health-promoting properties [7,32]. Derivatives of cyanidin, peonidin, malvidin, and pelargonidin have been detected in rice. Proanthocyanidins are found in red rice, comprising (+)-catechin and (–)-epicatechin monomeric structure units [7]. Red rice is rich in iron and zinc, while black and purple rice are rich in protein, fat, and crude fiber [2]. The effects of anthocyanins have been reported on antiproliferative activity, anti-inflammation, neuroprotective activity, anti-obesity, and antidiabetic activity [32], and prevention of cardiovascular disease [32,33].

Whole grain rice is known to have a higher phenolic content and stronger antioxidant capacity than polished rice [2]. In a previous study, ferulic acid, isoferulic acid, *p*-coumaric acid, and vanillic acid were identified in the insoluble bound fractions of the extract of rice flour obtained from a genetic cross rice between black and white rice parents [34]. Hydroxybenzoic and hydroxycinnamic acids, and flavonoids have been identified in brown rice [7]. Various types of flavonoids found in brown rice include flavonols, flavones, flavanols, flavanons, and isoflavones [7]. Relatedly, in the present study, protocatechuic acid (2), vanillic acid (5), and rutin (10) were identified in the methanolic extracts of RD6 and BGR sprouts.

Protocatechuic acid is the main metabolite of complex polyphenolic compounds such as anthocyanins and procyanidins [35,36,37]. Protocatechuic acid has long half-life in the blood at higher concentrations than parent compounds and can cross the blood brain barrier. Its reported pharmacological actions are strong antioxidant activity, anti-inflammation, antihyperglycemic activity, inhibition of chemical carcinogenesis, apoptosis induction, antiproliferative effects in different cancerous tissues, analgesic effect, and antiviral and antimicrobial activities [35,37,38]. Protocatechuic acid moreover has potential vis-à-vis prevention and/or treatment of neurodegenerative disorders including Alzheimer’s and Parkinson’s diseases. The underlying mechanisms were related to the cognitive and behavioral impairment such as accumulation of the β-amyloid plaques in brain tissues, hyperphosphorylation of tau protein in neurons, excessive formation of reactive oxygen species, and neuroinflammation [36].

Vanillic acid is one of the main catechins metabolites found in humans after consumption of green tea infusions [39]. It also occurs in the production of vanillin from ferulic acid [40]. Vanillic acid has been reported to exert anti-oxidation, anti-pain, anti-inflammation, and prevent neurodegenerative disorders [41,42,43]. The mechanism of action of vanillic acid included inhibition of cyclooxygenase-2 (COX-2) expression, decreasing prostaglandin E2 (PGE2) production, inhibition of receptor-interacting protein (RIP)-2/caspase-1 pathway, and nitric oxide (NO) production in mouse peritoneal macrophage cells [42,43]. Vanillic acid also inhibited phosphorylation of inhibitory kappa-B and decreased nuclear translocation of p65 resulting in the reduction of nuclear factor kappa-B activity and its consequent pro-inflammatory cytokines (e.g., tumor necrosis factor (TNF)-α, and interleukin (IL)-6) [41,42]. Vanillic acid also suppressed COX-2 levels in a mouse model of ulcerative colitis [41].

Rutin is also known as vitamin P or rutoside. Its structure includes a glycoside of flavonolic aglycone quercetin and disaccharide rutinose [44]. Rutin has various pharmacological effects [44] including anti-oxidant, cytoprotective effect, vaso-protective effect, anti-carcinogen, neuroprotection, cardio-protection [44], and anti-hyperglycaemic and anti-oxidant activity in streptozotocin-induced diabetic rats [45].

It should be noted that the difference in the types and amounts of compounds found between the current study and previous reports could be affected by several factors such as genetic make-up, harvesting time, part used, and growing condition of rice as well as the type of solvent used for the extraction [2,7]. The bioactive compounds are, moreover, synthesized after photosynthesis as a second metabolite and are later being transferred to other parts of the plant at a later stage of the life cycle. As a consequence, different plant parts will have different types and contents of compounds at various times in the life cycle of the plant.

In conclusion, to the best of our knowledge this is the first report on the influence of harvest time on phytochemicals and proximate compositions of the extract of rice sprouts of RD6 and BGR extracts. Our study provides information on the potential health benefits of rice sprouts and suggests the importance of harvest time on developing functional foods from rice sprout extracts. Further study is required regarding bioactivity and the clinical levels for safe consumption.

## 4. Materials and Methods

### 4.1. Chemicals

Analytical reagent grade methanol (from V.S. Chem House, Bangkok, Thailand) and hydrochloric acid (HCl) 37% (from RCI Labscan (Bangkok, Thailand)) in analytical reagent grade were used for the extraction. Acetonitrile and acetic acid both in HPLC grade were purchased from V.S. Chem House. Deionized water (DI H_2_O) obtained from PURELAB option–Q (High Wycombe, UK) was used for the HPLC analysis. The phenolic acid standards (i.e., gallic acid, protocatechuic acid, *p*–hydroxybenzoic acid, chlorogenic acid, vanillic acid, caffeic acid, syringic acid, *p*–coumaric acid, and ferulic acid) and the flavonoid acid standard (rutin) were purchased from Sigma–Aldrich Co. (St. Louis, MO, USA). Folin Ciocalteu reagent (Carlo Erba Reagenti, Milan, Italy), sodium carbonate (NaCO_3_) (Ajax Finechem Pty Ltd., Auckland, New Zealand) were used for the total phenolic analysis. Potassium chloride (KCl) and sodium acetate (CH_3_COONa·3H_2_O) were purchased from Ajax Finechem Pty Ltd. and were used for total anthocyanin analysis. Ethanol 95% (from ACL Lab Scan Co. Ltd., Bangkok, Thailand), and diethyl ether and petroleum ether were purchased from RCL Lab Scan Co.Ltd., while CuSO_4_ ·5H_2_O solution, K_2_SO_4_, H_3_BO_3_ solution were purchased from Merck, Darmstadt, Germany), and 50% NaOH (from ACL Lab Scan Co. Ltd., Bangkok, Thailand).

### 4.2. Plant Material and Extractions

The aerial part of rice (*Oryza sativa*) or sprouts from two cultivars—white (RD6) and black (BGR) glutinous rice—were harvested in October 2018 from a fertilizer-, pesticide-free farm in Khon Kaen province, Thailand. The rice was grown for the current study and collected for extraction for use throughout the study. The codes of the rice cultivar were assigned by the Bureau of Seed Multiplication, Rice Department of Thailand. The rice sprouts were collected at three different ages: 5–7 days (height, 7–10 cm), 10–15 days (height, 11–14 cm), and 20–25 days (height, 15–18 cm). The sprouts were washed and dried at room temperature before extraction and were cut into small pieces and sonicated with 1% HCl in methanol (20 g per 100 mL) for 30 min. Sequential maceration and sonication was done with 2 × 100 mL of solvent. The extract was filtered through filter paper. The solvent was removed by a rotary evaporator at a temperature below 45 °C and moisture was removed by a freeze drier to obtain the dry residue. The crude extracts stored under 4 °C until analyzed.

### 4.3. Identification of Phenolics and Flavonoids by Using HPLC

The component and content of phenolics and flavonoids in the extract of the aerial part of rice were determined by high performance liquid chromatography (HPLC). Phenolics and flavonoids in samples were identified by comparing the retention time and spectral matching to standards. The phenolic standards were gallic acid, protocatechuic acid, *p*–hydroxybenzoic acid, chlorogenic acid, vanillic acid, caffeic acid, syringic acid, *p*–coumaric acid, and ferulic acid. The flavonoid standard was rutin. Analyses were performed using an LC–2030C3D quaternary pump (Shimadzu, Kyoto, Japan) equipped with a diode array detector (DAD) according to the method by Butsat et al. [46] The extracts were dissolved in methanol (HPLC grade), filtered through a 0.45 μm membrane filter and injected onto a HiQ sil C18W column (4.6 mm × 250 mm, 5 μm) (KYA Technologies Corporation, Tokyo, Japan). The injection volume was 20 μL using an autosampler. A gradient of solvent A (1% acetic acid in water) and solvent B (acetonitrile) was used at a flow rate of 0.8 mL/min. The column temperature was set at 38 °C. The gradient elution was performed as follows: from 0 to 5 min, linear gradient from 5 to 9% solvent B; from 5 to 15 min, 9 to 11% solvent B; from 15 to 22 min, linear gradient from 11 to 15% solvent B; from 22 to 30 min, linear gradient from 15 to 18% solvent B; from 30 to 38 min, linear gradient from 18 to 23% solvent B; from 38 to 43 min, linear gradient from 23 to 80% solvent B; from 43 to 46 min, linear gradient from 80 to 90% solvent B; from 46 to 55 min, isocratic at 90–95% solvent B; from 55 to 60 min, linear gradient from 95 to 5% solvent B; and a re–equilibration period of 10 min with 5% solvent B used between individual runs. The detector wavelength was set at 280, 320 and 370 nm. The standard hydroxybenzoic acids were gallic acid, protocatechuic acid, *p*–hydroxybenzoic acid, vanillic acid, syringic acid). The standard hydroxycinnamic acids were chlorogenic acid, caffeic acid, *p*–coumaric acid, and ferulic acid. The standard flavonoids was rutin. Compounds in the samples were identified by comparing their relative retention times and UV spectral pattern with those of authentic compounds and were confirmed by using a co–injection of the standard with the extract sample.

### 4.4. Determination of Total Phenolic Contents 

Total phenolic content (TPC) was determined by the Folin–Ciocalteu method with some modifications [46,47,48]. Briefly, 15 µL of extract (10 mg/mL in methanol) was mixed with Folin–Ciocalteu reagent (120 µL). After 5 min, Na_2_CO_3_ solution at a concentration of 60 g/L (120 µL) was added. The solution was mixed and incubated in the dark for 90 min. The absorbance was measured at 725 nm by microplate reader against a blank (EnSight Multimode plate reader, Waltham, MA, USA) against a blank. The TPC of extract was calculated from the gallic acid standard curve (y = 0.0643x + 0.0267, R^2^ = 0.9997) and expressed as the gallic acid equivalent (GAE) in mg per gram of dry weight of the extract. Data represent the average of 5 replicates. 

### 4.5. Determination of Total Chlorophyll Contents 

Total chlorophyll content (TCC) was determined based on the method described by Özkan and Bilek [22]. Briefly, 200 µL of extract (10 mg/mL in methanol) was scanned at 645 and 663 nm by microplate spectrophotometer. The total chlorophyll contents was calculated by Equation (1). Data represent the average of the replicates:

Total chlorophyll content (mg per mg extract) = [(20.2 × A_645_) + (8.02 × A_633_)]/[1000] (1)

### 4.6. Determination of Total Anthocyanins Content

Total anthocyanin content (TAC) was evaluated by using modifications of the pH differential method [30,34,48,49] in a 96-well plate. Briefly, the two different pH solutions were prepared—viz., pH 1.0 potassium chloride buffer (pH 1.0 KCl buffer), and pH 4.5 sodium acetate buffer (pH 4.5 CH_3_CO_2_Na buffer). The extract in methanol was diluted 10 times with the two buffers, shaken under dark conditions for 15 min and then centrifuged at 2500× *g* for 10 min at room temperature before measuring of absorbance at 510 nm and 700 nm by microplate reader. Blank (DI water) determination was also conducted. Total monomeric anthocyanin of extract was calculated following Equation (2) in terms of cyanidin 3–glucoside. The results were expressed as mg of cyanidin 3–glucoside equivalent per gram of dry weight and the average of four replicates:

Total anthocyanin content (mg/g) = [A_diff_ × Mw × DF × 1000]/[ε] (2)

where A is (A_510_–A_700_) _pH 1.0_ − (A_510_–A_700_) _pH 4.5_, Mw is the molecular weight of cyanidin–3–glucoside (g/mol), DF is the dilution factor (10), ε is the molar extinction coefficient for 26,900 L mol^−1^cm^−1^.

### 4.7. Determination of Total Flavonoid Content

The determination of the total flavonoid content (TFC) of the extract samples was done as previously [50]. Quercetin was used as the standard to make a standard curve. Stock quercetin solution was prepared by dissolving 0.1 mg quercetin in 1.0 mL ethanol, then the standard solutions of quercetin were prepared by serial dilutions using ethanol (1–50 μg/mL). An amount of 100 µL diluted standard quercetin solutions or extracts was mixed separately with 50 µL of 2% aluminum chloride. After mixing, the solution was incubated for 60 min at room temperature. The absorbance of the reaction mixtures was measured against a blank at 415 nm wavelength with a microplate reader. The concentration of TFC in the rice sprout extract was extrapolated from the calibration plot of quercetin (y = 0.0449x, R^2^ = 0.995). The results were expressed as mg quercetin equivalent (QE)/g of dried extract. All the determinations were carried out in quadruplicate.

### 4.8. Proximate Analysis

#### 4.8.1. Determination of Total Ash

Fresh rice sprouts were ground and weighed (3 g) into broad ashing dishes. Samples were ignited in a furnace at 550 °C until a light gray ash was obtained which was cooled in a desiccator desiccator to get a constant weight. Total ash was calculated as follows [51]:

Total ash = (W_2_ − W_1_)/(W) (3)

where w is grams of sample, w_1_ is grams of the broad ashing dish and sample before combustion, w_2_ is grams of the broad ashing dish and sample after combustion. 

#### 4.8.2. Determination of Moisture

The fresh rice sprouts were ground into fine pieces and weighed (2 g) in a covered, tared aluminum dish. The rice sprouts in the covered container were dried in an oven at 130 ± 3 °C for 1 h then the sprouts were transferred to a desiccator to get a constant weight. The calculated %moisture content was calculated using the following equation [51]:

%Moisture = [100 × (W_1_ − W_2_)]/ [W_1_ − W] (4)

where w is the weight of the aluminum dish and cover, w_1_ is the weight of the aluminum dish, cover, and sample before drying, w_2_ is the weight of the aluminum dish, cover, and sample after drying.

#### 4.8.3. Determination of Fat

The ground fresh rice sprout (2 g) was weighed into a beaker and 2 mL of 95% ethanol was added and mixed well. Then 10 mL of 37% HCl was added and stirred at frequent intervals in water baht at 70–80 °C for 30 min. After left cooling at room temperature, the 10 mL of 95% ethanol was added. The mixture was transferred to a separatory funnel. The beaker was rinsed with 25 mL diethyl ether. Then 25 mL of petroleum ether was added into the separatory funnel and was shaken vigorously for 1 min. The sequential separation was done three times (25 mL × 3). The complete separation was observed when the liquid was practically clear. The petroleum ether layer was collected and transferred to beaker, which was previously dried in oven at 100 °C. The beaker was placed on water baht to evaporate ether. After that, it was cooled down at room temperature to get constant weight of fat, which was expressed as %fat and calculated using the following Equation (5) [51]:

%Fat = [100 × (W_2_ − W_1_)]/[W] (5)

where w is grams of sample, w_1_ is gram of the dish (rinsed with petroleum ether), w_2_ is gram of the dish and fat after drying.

#### 4.8.4. Determination of Protein

The fresh samples were spun into fine fibers and 1 g was weighed into a digestion tube containing K_2_SO_4_ and CuSO_4_·5H_2_O solution in a 9:1 ratio. Twenty mL of H_2_SO_4_ was then added to the mixture which was digested at 180–230 °C until the clear sample turned to a light blue-green color which was then digested (boiled) for another 1 h. The sample was transferred to cool at room temperature and 85 mL of water was added and well mixed by shaking. The digestion tube was then transferred into a distillation machine and 65 mL of 50% NaOH was added. The distillate in the distillation titration flask contained 50 mL H_3_BO_3._ The distillate was titrated with 0.1000 M HCl until a pink color was seen—the endpoint. Two different experiments were done in duplications. The protein content was calculated as following Equations (6) and (7) [51]:

Nitrogen (g/100 g) = [14.007 × M × (V_a_ − V_b_) × 100]/[W × 1000] (6)

Protein (g/100 g) = Nitrogen × 6.25(7)

where w is gram sample, M is the molarity of the HCl solution, V_a_ is the volume of HCl that used to titrate the sample, V_b_ is the volume of HCl that used to titrate the blank.

#### 4.8.5. Determination of Carbohydrate

Carbohydrate was calculated by the sum of percentages of protein, lipid, moisture and ash using the following Equation (8) then subtracted from 100. The result was expressed as %total carbohydrates [52]:

%Total carbohydrate = 100 − (%protein + %fat + %moisture + %ash)(8)

#### 4.8.6. Determination of Energy 

Energy was calculated based on the following Equation (9) and the value unit was represented as kcal/100 g [52]:

Energy (kcal/100 g) = (%protein × 4) + (%fat × 9) + (%carbohydrate × 4)(9)

#### 4.8.7. Determination of Dietary Fiber 

Dietary fibers analysis was performed according to AOAC (2016) 985.29 method [51]. The fresh samples were spun into fine fibers and phosphate buffer added and mixed well. The enzyme amylase was added to the solution and incubated at 95 °C for 30 min. After that the sample was cooled at room temperature. The sample was adjusted to pH 7.5 before adding the enzyme protease and incubated at 60 °C for 30 min for protein removal. The sample was cooled at room temperature and the pH adjusted to 4.5. The enzyme amyloglucosidase was added and the mixture incubated at 60 °C for 30 min for carbohydrate removal. 95% ethyl alcohol was added to get complete precipitation. The two experiments were done in duplicate. 

### 4.9. Statistical Analysis

Experimental results are presented as means ± standard deviation (SD) except for the proximate tests which were done in duplicate. Between group statistical analyses were performed using a one–way ANOVA followed by Tukey multiple comparison tests. Significant difference was set at *p*-value < 0.05. Regression and partial correlation analyses were performed using SPSS 19 (SPSS Inc, Chicago, IL, USA).

## Figures and Tables

**Table 1:** Detection wavelength and retention times of standard phenolic and flavonoid compounds (*n* = 3).

Standard Compounds	Retention Time (min)		
	280 nm	320 nm	370 nm
**Hydroxybenzoic Acids**			
Gallic acid (1)	5.74 ± 0.01	5.74 ± 0.01	ND
Protocatechuic acid (2)	8.58 ± 0.08	8.58 ± 0.08	ND
*p*-Hydroxybenzoic acid (3)	12.06 ± 0.03	ND	ND
Vanillic acid (5)	14.89 ± 0.02	ND	ND
Syringic acid (7)	17.19 ± 0.01	ND	ND
**Hydroxycinnamic Acids**			
Chlorogenic acid (4)	13.71 ± 0.03	13.71 ± 0.03	13.71 ± 0.03
Caffeic acid (6)	15.56 ± 0.03	15.64 ± 0.03	15.57 ± 0.03
*p*-Coumaric acid (8)	23.10 ± 0.07	23.10 ± 0.07	not detected
Ferulic acid (9)	27.29 ± 0.02	27.29 ± 0.02	27.24 ± 0.02
**Flavonoids**			
Rutin (10)	32.89 ± 0.06	32.89 ± 0.06	32.89 ± 0.06

ND = Not detected.

**Table 2:** Phenolic and flavonoid contents in the rice sprout extracts (RD6 and BGR) based on HPLC analysis.

Compound (Peak Number)	Detected Amount (mg/g) and Harvest Date					
	RD6			BGR		
	5−7 Days	10−15 Days	20−25 Days	5−7 Days	10−15 Days	20−25 Days
	7–10 cm	11–14 cm	15–18 cm	7–10 cm	11–14 cm	15–18 cm
**Hydroxybenzoic Acids**						
Protocatechuic acid (2)	1.58 ± 0.12 ^a,A^	1.31 ± 0.08 ^b,B^	0.38 ± 0.03 ^d,C^	1.44 ± 0.12 ^ab,B^	0.90 ± 0.10 ^c,B^	0.56 ± 0.01 ^d,B^
Vanillic acid (5)	1.45 ± 0.12 ^cd,A^	0.65 ± 0.06 ^d,C^	1.56 ± 0.14 ^c,A^	7.69 ± 0.58 ^a,A^	4.39 ± 0.48 ^b,A^	1.28 ± 0.01 ^cd,A^
Total	3.04 ± 0.24	1.96 ± 0.14	1.94 ± 0.17	9.12 ± 0.7	5.29 ± 0.57	1.84 ± 0.03
**Flavonoids**						
Rutin (10)	1.68 ± 0.11 ^a,A^	1.60 ± 0.10 ^a,A^	0.98 ± 0.08 ^c,B^	0.47 ± 0.04 ^d,C^	1.25 ± 0.08 ^b,B^	1.32 ± 0.01 ^b,A^
Total	1.68 ± 0.11	1.60 ± 0.10	0.98 ± 0.08	0.47 ± 0.04	1.25 ± 0.08	1.32 ± 0.01

ND = Not detected. Results are presented as the mean ± SD (*n* = 3). The different superscript letters between the harvest date of both cultivars in the same row (denoted by small letters) or between the compounds at the same harvest date in the same column (denoted by upper case letters) differ significantly (*p* < 0.05).

**Table 3:** Total phenolic content (TPC), total chlorophyll content (TCC), total anthocyanin content (TAC), and proximate composition of RD6 and BGR rice extracts.

Phytochemical or Component (Units)	RD6			BGR		
	5–7 Days	10–15 Days	20–25 Days	5–7 Days	10–15 Days	20–25 Days
	7–10 cm	11–14 cm	15–18 cm	7–10 cm	11–14 cm	15–18 cm
**Phytochemical content (mg) per g of dry extract (DE)**						
TPC (mg GAE)	44.8 ± 1.6 ^a^	41.5 ± 2.2 ^b^	29.1 ± 1.4 ^c,d^	28.1 ± 0.6 ^d^	31.9 ± 1.1 ^c^	24.9 ± 1.3 ^e^
TCC (mg)	40.5 ± 2.2 ^a^	34.6 ± 2.1 ^c^	35.9 ± 1.9 ^b^	19.3 ± 1.8 ^d^	40.8 ± 0.8 ^a^	40.6 ± 0.8 ^a^
TAC (mg C_3_GE)	0.06 ± 0.001 ^c^	0.01 ± 0.001 ^c^	0.15 ± 0.001 ^c^	22.13 ± 0.002 ^a^	0.45 ± 0.001 ^b,c^	0.71 ± 0.001 ^b^
TFC (mg QE)	34.2 ± 3.4 ^a^	32.6 ± 0.3 ^a,b^	16.3 ± 0.5 ^d^	21.5 ± 1.4 ^c^	30.3 ± 0.3 ^b^	29.2 ± 0.7 ^b^
**Proximate composition (g) per 100 g fresh weight**						
Carbohydrate	12.22	12.68	15.34	16.39	24.54	15.09
Protein	5.99	4.96	4.81	5.32	3.76	3.38
Fat	0.75	0.32	1.01	0.39	1.20	0.78
Ash	2.86	2.93	3.65	3.31	5.78	4.02
Moisture	78.18	79.11	75.19	74.59	64.72	76.73
Energy	79.59	73.44	89.69	90.35	124.00	80.90
Dietary fiber	nd	11.98	nd	nd	16.92	nd

Results of phytochemical contents are presented as the mean ± SD (*n* = 3−5). The different superscript letters differ significantly (*p* < 0.05). TPC = Total phenolics content (mg gallic acid equivalent (GAE)/g of dry extract), TCC = Total chlorophyll content (mg per mg extract), Total anthocyanin content (mg cyaniding-3-glucoside equivalent (C_3_GE) per g extract), Total flavonoid content (mg quercetin equivalent (QE)/g of extract), nd = not determined.

## Supplementary Materials

The following are available online, Figure S1. HPLC chromatograms of the mixture of 10 standard phenolics and flavonoids at 500 µg/ml in methanol detected at 280, 320 and 370 nm. Peaks for phenolics are (i) hydroxybenzoic acids (viz., gallic acid (1), protocatechuic acid (2), p–hydroxybenzoic acid (3), vanillic acid (5), syringic acid (7); and, (ii) hydroxycinnamic acids (viz., chlorogenic acid (4), caffeic acid (6), p–coumaric acid (8), and ferulic acid (9). The flavonoid peak is rutin (10). Table S1. Summary of R2 obtained from the linear regression analysis among the phytochemical content and between the phytochemical content and harvest age of the RD6 and BGR cultivars. Table S2. Summary of the correlation coefficient (r) of phytochemical content and harvest age of each.cultivar.
