# Supplementary material for: Harvest Age Effect on Phytochemical Content of White and Black Glutinous Rice Cultivars
Source: Molecules. 2019 Dec 4;24(24):4432. doi: 10.3390/molecules24244432 (PMC6943599; doi:10.3390/molecules24244432)
Supplement: Supplementary file 1 [file molecules-24-04432-s001.pdf]

Supplementary Materials

Article

Harvest Age Effect on Phytochemical Content of White and Black Glutinous Rice Cultivars

Kawintra Tamprasit [1], Natthida Weerapreeyakul [2,3,*] Khaetthareeya Sutthanut [2,3], Wipawee Thukhammee [3,4], Jintanaporn Wattanathorn [3,4]

[1] Graduate School (in the program of Aesthetic Sciences and Health), Faculty of Pharmaceutical Sciences, Khon Kaen University, Khon Kaen, 40002, Thailand; kawintra_pop@hotmail.com (K.T.)
[2] Division of Pharmaceutical Chemistry, Faculty of Pharmaceutical Sciences, Khon Kaen University, Khon Kaen, 40002, Thailand; natthida@kku.ac.th (N.W.); khaesu@kku.ac.th (K.S.)
[3] Human High Performance and Health Promotion (HHP&HP) Research Institute, Khon Kaen University, Khon Kaen, 40002, Thailand; natthida@kku.ac.th (N.W.); khaesu@kku.ac.th (K.S.); meewep@gmail.com
[4] Department of Physiology, Faculty of Medicine, Khon Kaen University, Khon Kaen, 40002, Thailand; meewep@gmail.com (W.T.); jinwat05@gmail.com (J.W.)
* Correspondence: natthida@kku.ac.th (N.W.); Tel.: +66-4320-2378

Received: date; Accepted: date; Published: date

<u>Supplementary Material, Figure S1</u>

(A) 280 nm

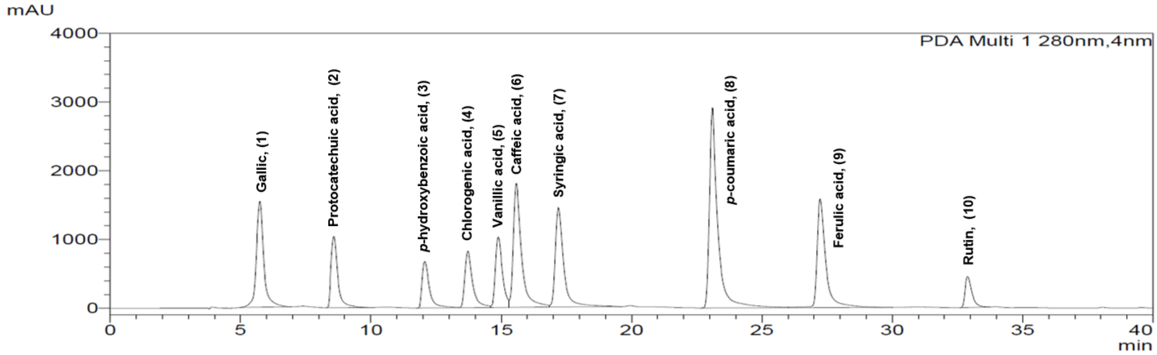

(B) 320 nm

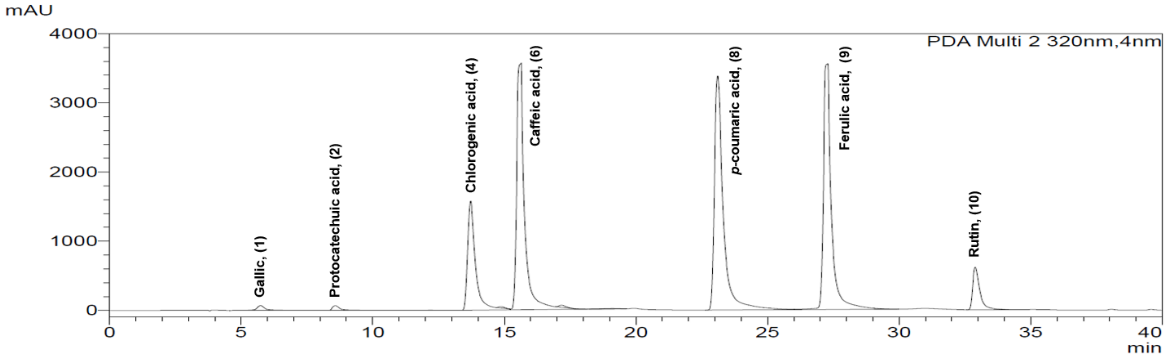

(C) 370 nm

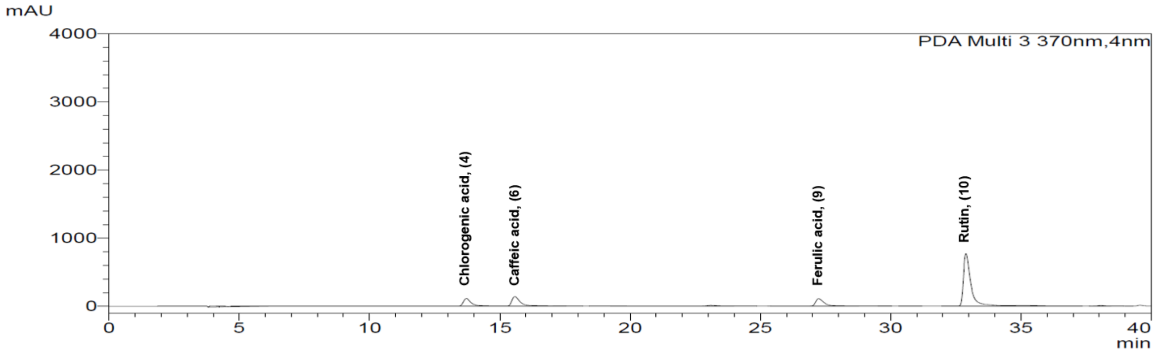

Figure S1. HPLC chromatograms of the mixture of 10 standard phenolics and flavonoids at 500 μg/ml in methanol detected at 280, 320 and 370 nm. Peaks for phenolics are (i) hydroxybenzoic acids (viz., gallic acid (1), protocatechuic acid (2), *p*–hydroxybenzoic acid (3), vanillic acid (5), syringic acid (7); and, (ii) hydroxycinnamic acids (viz., chlorogenic acid (4), caffeic acid (6), *p*–coumaric acid (8), and ferulic acid (9). The flavonoid peak is rutin (10).

Supplementary Material, Table S1

Table S1 Summary of R^2 obtained from the linear regression analysis among the phytochemical content and between the phytochemical content and harvest age of the RD6 and BGR cultivars**.**

			**BGR**			
			**TPC**	**TCC**	**TAC**	**TFC**
			0.185	0.745	0.774	0.626
**RD6**	**TPC**	**0.855**		0.011	0.012	0.044
	**TCC**	0.540	0.268		**1.000**	**0.962**
	**TAC**	0.112	0.214	0.002		**0.948**
	**TFC**	0.780	**0.931**	0.100	0.183	

The lower left values represent the correlation coefficient of the RD6 cultivar, while the upper right values represent those of the BGR cultivar.

Supplementary Material, Table S2

Table S2 Summary of the correlation coefficient (r) of phytochemical content and harvest age of each cultivar.

			BGR			
			TPC	TCC	TAC	TFC
			–0.396 (*p*=0.292)	0.863* (*p*=0.003)	–0.880* (*p*=0.001)	0.791* (*p*=0.002)
RD6	TPC	–0.944* (*p*<0.001)	1.000	0.107 (*p*=0.7884)	–0.111 (*p*=0.760)	0.210 (*p*=513)
	TCC	–0.735* (*p*=0.024)	0.518 (*p*=0.154)	1.000	–1.000* (p<0.001)	0.981* (*p*<0.001)
	TAC	0.335 (*p*=0.314)	–0.463 (*p*=0.152)	0.045 (*p*=0.908)	1.000	–0.974* (*p*<0.001)
	TFC	–0.883* (*p*<0.001)	0.965* (*p*<0.001)	0.316 (*p*=0.408)	–0.428 (*p*=0.189)	1.000

The lower left values represent the correlation coefficient of the RD6 cultivar, while the upper right values represent those of the BGR cultivar. TPC = Total phenolic content, TCC = Total chlorophyll content, Total anthocyanin content, TFC = Total flavonoid content, (r) near to +1 or −1 indicates a strong relationship and near to 0 indicates a weak or no relationship. *Correlation is significant at $p < 0.05$.
